# Homoeopathy

**Published:** 1845-11-01

**Authors:** 


*Homoeopathy.*—We believe it has come to be a generally acknowledged principle among medical men that the *laissez faire*, or let-alone policy is the best which they can pursue with reference to medical delusions, from time to time adopted and fostered by public credulity. Any formal or direct opposition is sure to lead to a misconstruction of motives. It is attributed to jealousy or self-interest, and the error or folly which we would unmask, derives confirmation and encouragement from our efforts to open the eyes of the community to the impositions practiced upon them. On this account we would forbear, both in our public and private intercourse with community, to attack any of the various schemes of Empiricism. All that we could desire to do is to define our position with regard to them. Farther than this any exertions are useless, since a palpable *reductio ad absurdum* would avail nothing in opposition to blind prejudice or conceited dogmatism.

Our brethren in other places may have some curiosity to know how the latest and most fashionable humbug, the title of which we have placed at the head of this article, thrives in this community. It was not to be expected that a town of this size would escape the visitation of this epidemic, which, like the Cholera, passes successively from one locality to another, following the course of navigable streams, rail roads and canals, dwelling for a season in all prominent thoroughfares. Its commencement in our city may be dated about eighteen months since. So far as the medical fraternity is concerned, the views which we have just expressed, have, we believe, been strictly followed. It has been allowed to have full scope to develope whatever results it could legitimately produce. For ourselves, when we have said aught on the subject, it has been to encourage trial of it by those already disposed to do so, rather than to endeavor to remove prepossessions in its favor. In so doing, we have had full confidence that by the absence of all restraint, as Shakespeare says of love sickness, "the appetite would sicken, and so die." We believe that its course has already advanced to the *sickening* stage, and that its final demise is not far distant.

Homœopathy, however, will doubtless be neglected only to be succeeded by some other contrivance to satisfy the inexhaustible appetency for the marvellous and ridiculous. Hydropathy appears now most likely to follow in its footsteps; and after this ephemeral absurdity has had its day, others will spring up to suit the progressive taste of that portion of society who will never be satisfied with plain, substantial, every-day wisdom.

While human nature remains unchanged, and until medicine becomes a demonstrative science, it is to be expected that such delusions will prevail to a greater or less extent. As medical men we may do much indirectly toward limiting their diffusion, by enlightening society on the subject of Physiology, by illustrating the true position of medical science, and the conditions of its successful prosecution. We can, also, do much by elevating the standard of medical acquirements, and by the constant improvement of our profession. Since persons naturally judge of a pursuit by its professors, it is no doubt one reason why many think disparagingly of the medical profession that there are so many embraced within it who are poorly qualified to represent its actual condition. After discharging our duty in these respects, it behooves us to exercise forbearance and charity in view of the fact, that folly, and injustice are inevitably incident to human weakness and frailty.

